# The recovery process across the menstrual cycle in recreational female athletes: a prospective cohort study

**DOI:** 10.1007/s11332-025-01552-1

**Published:** 2025-09-24

**Authors:** E. Nygaard Parsons, J. J. Mitchell, N. Mallon, G. Bruinvels, J. M. Blodgett

**Affiliations:** 1 Institute of Sport, Exercise & Health, Division of Surgery, and Interventional Science, UCL, 170 Tottenham Court Road, London, W1T 7HA UK; 2 UCL Medical School, UCL, London, UK; 3 Business Innovation Centre, Orreco Ltd., NUI Galway, Unit 103, Galway, Ireland; 4 Department of Tourism and Sport, Atlantic Technological University, Donegal, Ireland; 5 University College London Hospitals NIHR Biomedical Research Centre, London, UK

**Keywords:** Hs-CRP, Inflammation, Sport, Menstruation, Recovery

## Abstract

**Purpose:**

The relationship between post-exercise inflammation and menstrual cycle (MC) phases in female athletes is poorly understood. This study assessed recovery, measured via high-sensitivity C-reactive protein (hs-CRP), after game days in recreational female athletes and whether it differed by MC phase.

**Methods:**

Nineteen participants provided demographic and MC data and underwent point-of-care blood testing for hs-CRP on game day (GD) − 1, + 1, + 2, and + 3 on two occasions (*n* = 119 data collection days). Four MC phases were estimated using ovulation tests and self-reported bleed data. Random-effects regression models examined associations between GD and hs-CRP, and possible interactions between GD and MC. Backwards stepwise regression included the covariates; age, sport type, fatigue level, minutes played, MC symptoms, and BMI.

**Results:**

Compared to baseline (GD-1), hs-CRP was 25.0% (95% confidence interval:12.49,37.45) higher on GD + 1, 10.83% (− 1.66,23.31) higher on GD + 2 and returned to baseline by GD + 3 (− 3.20%(− 15.78,9.39)). Results were robust in adjusted models. A significant interaction between gameday and MC phase on hs-CRP revealed a 62.9% larger GD + 1 peak (40.31, 85.66) than GD-1 during the late luteal (LL) phase.

**Conclusion:**

Hs-CRP peaked on GD + 1, remained elevated on GD + 2, returned to baseline by GD + 3, with evidence suggesting inflammation was higher on GD + 1 in the LL phase.

**Supplementary Information:**

The online version contains supplementary material available at 10.1007/s11332-025-01552-1.

## Introduction

Over the past decade, participation in female sports has risen significantly, with many athletes attaining professional status [1]. Despite this, research focusing on optimising and understanding performance in female athletes remains limited, with only 6% of sport science research exclusively examining females [2]. This gap underscores the need for specific data to better understand how different aspects of sports performance, including recovery, can be refined to enhance performance and reduce injury risk.

As female sport becomes more professional, the demands of intensified training regimens have increased, which may contribute to elevated levels of acute inflammation, which can subsequently impact recovery times and injury [3, 4]. For example, the 2023 FIFA Women’s Benchmarking Study reported that daily training sessions are common, with player load averaging 14.9 h/week [5]. This is a significant increase from previous training programmes of approximately six hours/week prior, when players often trained 2–3 times weekly whilst balancing additional employment or education [5]. Increased training loads have been shown to play a role in the development of musculoskeletal conditions such as tendinopathy and muscle strains, as persistent inflammation can impair muscle recovery, increasing susceptibility to strains and prolonged healing times [6].

Given this increased physical load, gaining a better understanding of how female hormones may influence the recovery processes is essential for optimising performance and health. Studies in male athletes show that strenuous exercise can lead to exercise-induced muscle damage, which triggers an inflammatory response, peaking at 24 h, and lasting up to 48–72 h [3, 7–9]. There is limited data revealing this same trajectory among female athletes. A systematic review of 465 female football players (n = seven studies) found a similar pattern, with inflammation peaking at 24 h and returning to baseline by 72 h [10]. However, this study did not assess the effects of the menstrual cycle (MC) on the inflammatory response. Other studies have reported conflicting results, with Hackney et al. finding interleukin-6 (IL-6) levels significantly greater in the mid-follicular than mid-luteal phases post-exercise [11] and Silva et al. reported significantly greater IL-6 concentrations in the mid-luteal phase following exercise [12]. These conflicting findings underscore the need for further research using reliable systemic inflammation, and the present study seeks to help address this gap.

The MC may influence the recovery process, potentially explaining sex-specific differences during different MC phases in high-sensitivity C-reactive protein (hs-CRP) trajectories and recovery [13]. Females tend to have higher baseline hs-CRP levels than males, which may be influenced by hormonal factors such as oestrogen and variations in fat distribution [14].

The MC is characterised by hormonal and inflammatory activity fluctuations, with oestrogen and progesterone known to be regulators of inflammation [13, 15]. Oestrogen levels gradually rise during the early to mid-follicular (EF) phase (i.e. menstrual phase), peak in the late follicular (LF) phase and remain elevated throughout the mid luteal (ML) phase, before declining during the late luteal (LL) phase [16]. Progesterone levels are low during the EF and LF phases, before rising sharply during the ML phase and dropping during the LL phase, which is linked with increased inflammatory levels [17]. Therefore, the EF and LL phase, both characterised by lower oestrogen and progesterone levels, may hinder recovery due to increasing inflammation independent of exercise effects [18–20]. Despite these studies, there is limited evidence exploring whether hormonal fluctuations influence hs-CRP levels and their role in recovery following match-play in female athletes [21, 22].

To address these gaps, this study aimed to (i) characterise the post-game recovery process using hs-CRP levels in recreational female athletes and (ii) explore how the recovery process may change across the MC. Understanding these dynamics is essential for supporting female athletes. If recovery is found to be hindered at certain times in the MC, club staff and practitioners may be better informed to adjust training loads or implement interventions, such as player rotations and tailored nutrition strategies, to optimise player availability, while also improving overall wellbeing [23].

## Methods

### Study sample and design

A total of 20 recreational female athletes from eight sports (1 × athletics, 1 × basketball, 1 × cycling, 7 × football, 3 × netball, 2 × lacrosse, 2 × rowing, 3 × hockey) were recruited through an online campaign, posters, and email outreach. The inclusion criteria were restricted to: regularly cyclical, healthy female participants aged 18–35, engaging in weekly team-sport matches or individual sports fixtures and were classified as at least tier 2 (trained/developmental athletes) according to the Participant Classification Framework [24]. Individuals aged below 18 or above 35 years of age, those using hormonal contraceptives, and those who were pregnant, breastfeeding, or post-menopausal were excluded from the study. Informed consent was obtained from all participants before partaking in the study. Of the 20 participants, one was excluded from all analyses as they were ill during the study period, with high levels of acute inflammation. A further three were only included to address aim (i), as the MC phase could not be determined due to anovulatory cycles (*n* = 2) or no reported bleed phase (*n* = 1) during the study. Ethical approval was given by the UCL Research Ethics Committee (Project ID number: 25759/002).

**Fig. 1:**
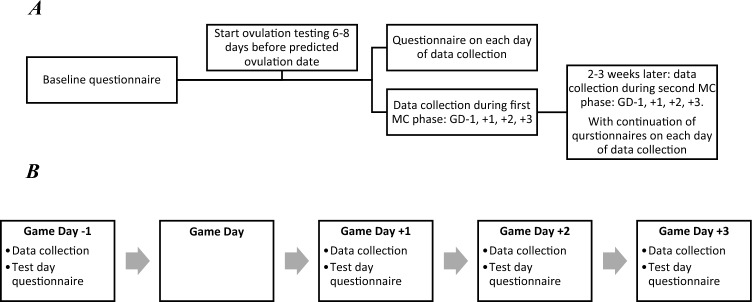
**A** An overview of the study design, consisting of the baseline questionnaire, ovulation testing protocol, data collection, and the test day questionnaire. **B**. An overview of the data collection protocol on GD-1, + 1, + 2 and + 3, which is repeated 2–3 weeks after

A prospective study design enabled repeat hs-CRP measurements to be collected on gameday (GD) -1, + 1, + 2 and + 3, for each monitored match or sports fixture, with participants undergoing two matches or sports fixtures in total. A repeat collection ~ 2 weeks later ensured data captured multiple MC phases within each individual (Fig. 1). Baseline inflammatory status was established based on the GD-1 data collection; data were not collected on game day to prevent confounding by potential hs-CRP elevation due to anxiety or adrenaline release, as well as the practicality of not interfering with pre-match preparation. Before data collection, participants completed a baseline questionnaire that gathered information on their weight, height, MC characteristics and current recovery routines. On each day of data collection, participants completed a test-day questionnaire to capture MC symptoms, play time and other relevant covariates (see more details in Fig. 1; see Supplemental Table 1, Supplemental digital content).

### Hs-CRP status assessment

Hs-CRP was selected as a reliable biomarker of inflammation due to its high sensitivity in measurement, allowing for the detection of subtle CRP increases [25, 26]. A point-of-care (POC) test was used to measure hs-CRP concentration, in which a 5μL finger-prick blood sample was obtained using a small lancet and analysis was conducted at room temperature, with an immunoturbidimetric hs-CRP assay on the cube-S POC analyser *(Eurolyser Diagnostica GmbH, Salzburg, Austria)* [27]. The Eurolyser, a latex-based photometric measuring device, determines hs-CRP concentrations through an antigen–antibody reaction between hs-CRP in the blood and anti-CRP antibodies bound to polystyrene latex particles [28]. This method demonstrated a negative predictive value of 93.8% [29]. The *Cube Eurolyser* exhibits good analytical performance, particularly in low haemoglobin concentrations and has low susceptibility to flaws in the POC procedure [30, 31]. To standardise the assessment of systemic inflammation, participants were instructed to report fasted, at a consistent time of day (i.e. first thing in the morning) [32].

### Menstrual cycle phase

MC phases were established using the participant’s self-reported menstruation data in combination with urinary ovulation kits. During the baseline questionnaire and each testing day, individuals reported their most recent menstruation start and end date using calendar counting and applications on mobile phones as the primary method to collect data. At least one MC was observed during the study. Ovulation testing began 6–8 days before the predicted ovulation date, calculated based on the individual’s last reported menstrual period. For cycle lengths between 22 and 24 days, ovulation testing started on day six (where day one is the first day of menstruation). For cycle lengths between 25 and 27 days, testing started on day 7, while for cycle lengths of 28 days or longer, testing began on day eight [33]. Ovulation testing was conducted daily until peak ovulation levels were detected, or for a maximum of ten consecutive days without a peak. Participants submitted photo evidence of each daily result (low, high or peak). The Clearblue Digital Ovulation Test is a quantitative hormone assay with accuracy exceeding 99% [34]. The ovulation test detects changes in luteinising hormone (LH) and estrone-2-glucuronide using monoclonal antibodies and eliminates subjective interpretation of results due to the device’s integrated digital reader [35, 36]. Testing was conducted in the morning, with participants instructed to conduct the urine tests as soon as possible upon waking, to align with the typical onset of the LH surge when urine is most concentrated following sleep [37].

To estimate the phase, a regression equation was used to predict the durations, previously defined by McIntosh et al. [38]. The phases were categorised as follows: EF phase was the menstruation period, LF phase was the remainder of the follicular phase up to and including peak ovulation, ML phase started after ovulation and constituted the majority of the luteal phase and the LL phase was the final five days preceding menstruation [34–37].

### Covariates

Covariates were selected based on prior research examining inflammation after exercise and symptoms were based on their common occurrence and impact on sporting performance [32, 39–43]. *Sport type* was categorised as contact (coded as 1) or non-contact (coded as 0). *Recent injury* was coded as 1 and 0 if no injury within the preceding three months. *MC symptoms* (4 symptoms encompassing bloating, nausea, lower back pain and cramps) and *fatigue* (3 symptoms encompassing heavy legs, muscle-ache, or weakness) were coded as 1 for the presence of any symptom and 0 for the absence of all symptoms; these were grouped due to the low prevalence across both individual symptom and symptom severity. *Fasting status* was coded as 1 if the participant had not eaten before data collection and 0 if they had. All covariates were assessed via self-reported test day questionnaires completed by the participants and were asked to specify if they had or had not experienced any of the symptoms listed (e.g. binary yes/no response). Continuous covariates included *minutes played*, *body mass inde*x (BMI) and *age*. Minutes played were determined by asking the participants to track their playing time and report it in the test day questionnaire. Participants maintained their usual training routines throughout the study, with most completing one competitive match or sports fixture per week, accompanied by one or two training sessions. *NSAIDs use within the last 24 h (yes/no)* was selected a priori as a covariate due to the acute effect on hs-CRP [44]; however, no participants reported NSAID use in the test day questionnaire.

### Statistical analyses

Descriptive statistics are presented as mean ± standard deviation (SD) or median (quartile 1, quartile 3) for continuous data, and frequencies and proportions for categorical data. Welch’s two-sample t-tests were used to compare the continuous variables, and Fisher’s Exact test was used to compare the categorical variables between the excluded and included groups, and differences in repeat-measure covariates across MC phase. Hs-CRP was not normally distributed as assessed using the Shapiro–Wilk test; therefore, hs-CRP levels were transformed to sympercents (*y* = 100 log(e)x) in regression models, with effect estimates indicating percent differences in hs-CRP compared to GD-1 (reference group) [45].

Random-effects models were used to account for both individual differences and overall trends in hs-CRP levels [46]. By estimating intercepts for each participant, the model accounted for individual variance in hs-CRP levels beyond what could be accounted for by fixed effects (between-participant variance) alone. Due to convergence issues, intercept-only random-effects models were used to analyse the association between GD and hs-CRP levels. To address aim (i), unadjusted models assessed the relationship between gameday and hs-CRP, followed by adjustments for covariates using a backwards stepwise regression approach. Next, to determine if a positive relationship between gameday and hs-CRP varied depending on the MC phase, an interaction term (gameday*MC phase) was added to both unadjusted and adjusted models. Where interactions were significant, models were re-centred at each phase to estimate hs-CRP levels across the recovery trajectory. All analyses were conducted using R studio (R Studio, Boston, Massachusetts, USA, 2020). A *p*-value < 0.05 was considered statistically significant.

## Results

### Sample characteristics

A total of 20 participants were included in the baseline characteristics. For aim (i) an analytic sample of 19 (aged: 18–32 years) was included, as one participant was excluded due to illness. A total of 16 participants were included in aim (ii) analysis with three participants excluded (*n* = 2 as the MC phase could not be determined for two participants as they experienced anovulatory cycles during the study and *n* = 1 had no reported bleed phase during the study period).

Table 1 provides baseline questionnaire covariates and game day covariates. Briefly, the average age and BMI of participants were 21.3 ± 2.87 years and 22.1 ± 1.54 kg/m^2^, respectively. The majority of participants were White (*n* = 15; 78.9%) and played a team-based contact sport (*n* = 16; 84.2%). There were no differences in age, BMI, sport type, menstrual cycle symptoms, fatigue, or injury status between the 16 participants included in the main analyses and the four excluded participants (see Supplemental Table 2, Supplemental Digital Content). However, those who were excluded were notably more likely to be from non-White ethnic backgrounds (75% vs 10%). Exploration of repeated measures covariates across MC phase indicated no differences in minutes played, acute injury/illness or fatigue levels across phases, however, the prevalence of MC symptoms was highest during the EF (68.0%) and LL (60.0%) phases, and lowest during the LF (20.0%) and ML (20.5%) phases (see Supplemental Table 3, Supplemental Digital Content).

**Table 1:** Baseline characteristics of participants (*N* = 19)

Baseline questionnaire covariates		
Age	Mean, SD	21.3 ± 2.87
BMI	Mean, SD	22.1 ± 1.54
Ethnicity		
White	% (N)	78.9% (15)
Asian	% (N)	21.1% (4)
Sport type^1^		
Contact Sports	% (N)	84.2% (16)
Non-Contact Sports	% (N)	15.8% (3)
Injury within the last 3 months		
Yes^2^	% (N)	10.5% (2)
No	% (N)	89.5% (17)

Game day covariates		
Fatigue^3^		
Yes	% (N)	51.5% (70)
No	% (N)	48.5% (66)
Menstrual cycle symptoms^3^		
Yes	% (N)	35.3% (48)
No	% (N)	63.0% (88)
Fasted^3^		
Yes	% (N)	87.5% (119)
No	% (N)	12.5% (17)
Injury during the study^3^		
Yes	% (N)	22.8% (31)
No	% (N)	77.2% (105)
Minutes Played	Mean, SD	59.9 ± 20.9

Contact sports – football, hockey, netball, lacrosse, basketball; non-contact sports – athletics, cycling, rowing

^2^The two injuries were a calf strain (unable to play for one week) and shoulder pain (able to play through injury)

3Coded for as binary (1 = presence of symptoms/fasted state, 0 = no presence of symptoms/not fasted)

### Hs-CRP and game day

**Fig. 2:**
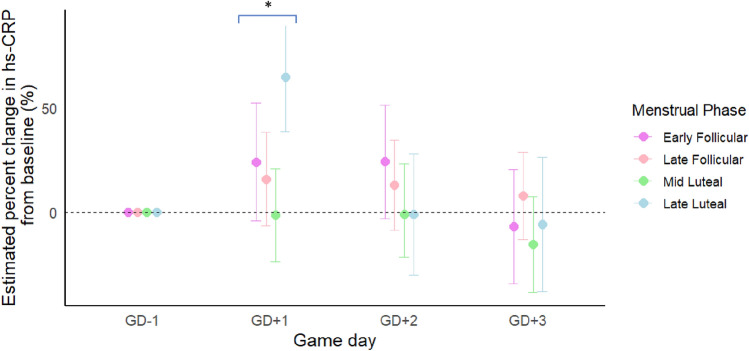
A forest plot showing the estimated percent changes in high-sensitivity C-reactive protein (hs-CRP) for each menstrual cycle phase, across game day (GD) *p < 0.05

Among the 19 participants included in aim (i) analyses, 14 provided complete data across two game day cycles and five participants contributed data for one game cycle, resulting in a total of 33 complete game day cycles. The data collected occurred across all MC phases: phase 1, *n* = 9 (20.9%) game day cycles; phase 2, *n* = 14 (32.6%); phase 3, *n* = 12 (27.9%) and phase 4, *n* = 8 (18.6%). Hs-CRP concentrations were shown to be non-normally distributed with a strong right skew (see Fig. 1, Supplemental Digital Content), (*p* < 0.001). Median hs-CRP levels increased from 0.73 mg/L (Q1:0.73, Q3:0.80) at GD-1 to a peak of 0.89 mg/L (Q1:0.77, Q3:1.16) at GD + 1. Levels decreased to 0.80 mg/L (Q1:0.75, Q3:0.90) at GD + 2 and 0.75 mg/L (Q1:0.71, Q3:0.80) at GD + 3 (see Fig. 2, Supplemental Digital Content). Compared to GD-1, adjusted models suggested that hs-CRP was 25.00% (95% confidence interval (CI): 12.69,37.60) higher on GD + 1, 10.83% (-1.63,23.28) higher on GD + 2, with no significant difference on GD + 3 (− 3.30% (− 15.78 – 9.39)) (see Table 2). In the backwards step-wise regression, age and participation in contact sports were associated with increased hs-CRP levels and remained in the adjusted model.

**Table 2:** Random-effect model results indicating changes in high-sensitivity C-reactive protein (hs-CRP) across game day (GD) (*n *= 19)

	Unadjusted model			Adjusted model^1^		
Predictor	Estimate	95% CI	P-value	Estimate	95% CI	*P*-value
GD-1 (ref)	–	–	–	–	–	–
GD+1	24.97	12.99–36.68	<0.01	25.00	12.49 – 37.45	<0.01
GD+2	10.83	− 1.15–22.81	0.08	10.83	− 1.66 – 23.31	0.09
GD+3	− 2.59	− 14.69–9.49	0.68	− 3.20	− 15.78 – 9.39	0.62

^*^CI Confidence interval, *SE* Standard error

^1^Adjusted for sport type and age

### Associations between hs-CRP and cycle phase and game day

When exploring interactions between game day and menstrual cycle phase, there was evidence of an interaction between the LL phase and GD + 1 (*p* = 0.04), indicating that inflammation was higher on GD + 1 during this phase compared to all other phases. For example, in adjusted models and compared to GD-1, hs-CRP was 64.65% (95% CI: 38.80,90.49) higher on GD + 1 during the LL phase. Conversely, hs-CRP was 24.20% (-3.96, 52.35), and 15.93% (− 6.45, 38.32) higher on GD + 1 in the EF and LF phases than GD-1, respectively, with no notable change in the ML phase (1.27% (− 23.63,21.10)) (see Fig. 2). No other MC phases demonstrated significant changes across game days, and no phase-specific differences were evident on GD + 2 or GD + 3. Complete estimates, including interaction terms and estimates with phase recentred are provided in Supplementary Digital Content Tables 4 and 5.

## Discussion

Findings suggest that post-game inflammation, as measured by hs-CRP concentrations, peaks on GD + 1, remains elevated at GD + 2, and returns to baseline by GD + 3 compared to GD-1. This recovery trajectory was influenced by the MC phase, with inflammation levels reaching a significantly higher peak on GD + 1 (64.65%, *p* < 0.05) during the LL phase. However, there were no differences in inflammation between phases on GD + 2 and GD + 3 suggesting that the MC may only influence the acute inflammatory response but not the overall timeline for recovery. These findings provide novel evidence suggesting inflammation levels during the acute post-game recovery period may be influenced by the MC phase. The results highlight the potential need to consider training load management, with greater emphasis on techniques to accelerate recovery during specific phases to optimise performance and potentially minimise injury risk.

### Comparison with previous research on hs-CRP in female athletes

The hs-CRP trajectory observed in this study, peaking within 48 h and returning to baseline by 72 h, is consistent with previous research examining inflammatory responses in female athletes. A systematic review reviewing 465 female football players across seven studies reported a moderate and significant increase in blood hs-CRP concentrations immediately post-match [10]. Within this review, six studies indicated that hs-CRP levels remained significantly elevated 24 h post-match, with a smaller but persistent effect observed at 48 h in five trials. By 72 h post-match, hs-CRP levels had largely returned to baseline, closely aligning with the pattern observed in this study. However, these studies did not compare differences in hs-CRP across MC phases [10]. The systematic review was limited by methodological heterogeneity, with small sample sizes, varied playing levels and inconsistent measurement timings that required broad recovery categories (e.g. ranges 0-1 h post-match, 1-12 h, 13-24 h, 25-48 h 49-72 h range), masking more precise kinetics [10]. Other studies have examined phase-related responses using hormonal verification [11], but findings report conflicting MC phases where inflammatory responses are the highest [11, 12]. In contrast, our study applied a controlled prospective design with repeated hs-CRP measurements at standardised time points across two matches per participant. MC phase was determined using self-reported data combined with urinary ovulation testing allowing us to examine phase-specific recovery kinetics.

### Recovery mechanism

Although research on female athletes’ performance remains limited, existing evidence highlights the impact of hormonal fluctuations across the MC on athletic performance, muscle strength and recovery [16]. Recovery, commonly defined as the return of performance measures to baseline [39], can also be indicated by the resolution of exercise-induced muscle damage, reflected by inflammatory biomarkers such as hs-CRP. In this study, recovery was defined as the return of systemic inflammatory markers, hs-CRP, to baseline [39]. Exercise-induced muscle damage triggers an inflammatory response, with rapid rises in hs-CRP and interleukin-6 levels, driven by mechanical and metabolic stress [47]. Within 24 h, damaged tissues are mostly phagocytosed, increasing pro-inflammatory cells, reducing inflammation, promoting tissue repair, and releasing cytokines like CRP [48]. CRP, synthesised by the liver, amplifies the inflammatory response by stimulating pro-inflammatory cytokines, which support the repair of microscopic muscle fibre damage, typically within 48–72 h [48].

The recovery timeline in this study aligns with this, suggesting that hs-CRP levels of female athletes return to baseline within 48–72 h. However, there was individual variability in hs-CRP concentrations of the athletes in the present study. This may indicate individualised adaptation to exercise, minimal muscle damage, or insufficient exercise intensity to trigger an inflammatory response [48]. Nevertheless, other inflammatory markers such as tumour necrosis factor and interleukin-10 fluctuate across the MC and can impact performance and recovery [49]. Additionally, ongoing metabolic stress and hormonal fluctuations during the MC may persist even when hs-CRP returns to baseline, highlighting the complexity of recovery beyond inflammation [17].

### Menstrual cycle phase variations in hs-CRP elevations

The intricate interplay between female sex hormones, particularly oestrogen and progesterone, throughout the MC is widely assumed to influence various aspects of performance and recovery [16, 18]. Findings of the present study suggest a trivial trend towards higher elevations in hs-CRP on GD + 1 during the LL phase. The LL phase has been linked to greater exercise-induced muscle damage and impaired recovery due to higher blood lactate concentrations compared to other phases, suggesting phase-specific differences in metabolic responses [50].

During phases characterised by lower oestrogen concentrations (e.g. EF and LL phases), recovery following strenuous exercise may be hindered. This could lead to a different inflammatory trajectory or with hs-CRP rising to higher levels than in the LF and ML phases, as seen in this study.” Although both the EF and LL phases have low oestrogen, their hormonal environments differ. The EF phase has low levels of both oestrogen and progesterone, while the LL phase is marked by a sharp decline in progesterone following its ML peak [17]. These hormonal fluctuations are known to influence immune function and inflammatory responses, with lower oestrogen and rapidly falling progesterone linked to reduced anti-inflammatory effects, which may partly explain the increased hs-CRP observed in the LL phase. [13, 17].

Physical and psychological MC symptoms can also impact recovery and inflammation, with evidence showing that fatigue, cramps, poor sleep quality and increased stress levels can impact hs-CRP [51, 52]. During the LL phase, when progesterone levels drop, leading to increased prostaglandin synthesis, causing cramps, and increased inflammatory responses [53]. Acute psychological declines can further disrupt sleep, increase fatigue and elevate stress [54]. The significant increase in MC symptoms during the EF and LL phases, compared to the LF and ML phases supports this. However, the observed associations remained after adjustment for MC symptoms, suggesting additional sources of peripheral inflammation downstream. These findings underscore the importance of managing training load and intensity in alignment with the current MC phase to optimise recovery and performance.

### Implications for practice and research

As an immediate next step, further research is needed on inflammation, recovery, and performance optimisation in female athletes, incorporating larger sample sizes, hormonal monitoring, detailed MC phase data, and performance-related measures to complement physiological markers and reflect the definition of recovery as a return to baseline. This would provide a more comprehensive understandings of how hormonal fluctuations influence recovery over time. Additionally, exploring individualised recovery strategies tailored to MC phases and specific inflammatory profiles could lead to the implementation of targeted interventions, such as training load management to ensure optimal athlete availability, recovery, and nutrition protocols. The fundamental aim is to keep players healthy and performing optimally on the pitch, therefore, these strategies could help minimise excessive inflammation. Further research should explore the impact of hormonal contraceptives on recovery and performance, as they can serve as a control for non-users, as they result in lower endogenous and higher exogenous sex hormone concentrations [55].

There is growing recognition of the need for research-driven approaches of female athlete health. Further evidence into how recovery My differ across the MC phase, and how this may differ by individual characteristics, could aid the development of more targeted recovery protocols across time and other characteristics (e.g. age, sport, ethnicity etc.).

### Strengths and limitations

Strengths of this study include the collection of objective blood biomarker recovery data across MC phases and game days. The CUBE Eurolyser provided accurate and reliable hs-CRP measurements. Comprehensive data collection, facilitated by detailed questionnaires and multiple post-match hs-CRP measurements, enabled in-depth analysis of hs-CRP variations. Additionally, each participant was assessed across at least one complete MC. This design allowed both within- and between-individual comparisons, enhancing understanding of factors influencing hs-CRP.

However, there were several limitations. No power calculation was performed as no previous study has explored this question and thus no effect size was available. It is likely that statistical power was limited, which may explain why inflammation at GD + 2 did not reach significance. Future studies should include larger, more homogenous samples (e.g. multiple football teams) to allow adequately powered analysis. Secondly, the real-world nature of field-based research limited control over game load, influencing inflammatory responses. Variability was also introduced by including athletes from different sports with differing match demands, as the research team did not have control over each individual’s game load; minutes played were included as a covariate. MC phase classification relied on self-reported bleeding and urinary ovulation test, which may have affected accuracy; direct measurement of blood oestrogen and progesterone could improve precision. Participants reported the first day of menses for each MC using calendar tracking. This non-invasive and feasible approach allowed repeated measures without the need for hormonal assays. However, calendar tracking may not fully capture hormonal fluctuations, particularly in participants with irregular cycles, potentially contributing to variability in hs-CRP and inconsistencies in inflammatory responses across the MC. Despite these limitations, calendar tracking combined with ovulation testing provided a practical method for tracking the MC in a real-world athletic sample. Some participants may not have experienced sufficient exercise-induced muscle damage, affecting the inflammatory response, whereas professional athletes may incur greater exercise-induced damage due to the higher intensity of competitive matches. Another limitation was that not all athletes reported in a fasted state. Although infrequent, this could have elevated CRP levels as the consumption of high-fat foods is known to increase inflammation and as such, this was controlled for in the main models. Finally, in this study, only one inflammatory marker was used, therefore, future research should incorporate other inflammatory markers like interleukin-6 and tumour necrosis factor-alpha for a more comprehensive analysis [56].

## Conclusion

This study found significant increases in hs-CRP on GD + 1, highest during the LL phase, with non-significant elevated levels on GD + 2 and a return to baseline by GD + 3 in amateur female athletes. Recovery varied by MC phase, with higher levels of inflammation during the LL phase. However, inflammation levels on GD + 2 and GD + 3 were similar across phases, indicating that while the MC may influence the initial inflammatory response, it may not substantially impact overall recovery time in this cohort. Future studies with larger sample sizes are needed to explore this relationship further. If supported by further research, these findings have practical implications, enabling teams to tailor recovery strategies by adjusting training loads and implementing targeted nutritional or pharmaceutical interventions based on an athlete’s MC phase.

## Supplementary Information

Below is the link to the electronic supplementary material.

Supplementary file1 (DOCX 123 KB)

## Data Availability

No datasets were generated or analysed during the current study.
