# Supplementary material for: The recovery process across the menstrual cycle in recreational female athletes: a prospective cohort study
Source: Sport Sci Health. 2025 Sep 24;21(4):3367–76. doi: 10.1007/s11332-025-01552-1 (PMC12647198; doi:10.1007/s11332-025-01552-1)
Supplement: Supplementary file 1 — Supplementary file1 (DOCX 123 KB) [file 11332_2025_1552_MOESM1_ESM.docx]

| **Supplemental Table 1.** Survey questions and response options used for covariates | | |  |
| --- | --- | --- | --- |
| **Question** | **Response Options** | **Coding** | |
| How old are you? | (In years) | Continuous | |
| What is your ethnic group? | Choose one option based on Office for National Statistics guidelines: White, Black/African/Caribbean/Black British, Asian/Asian British, Mixed/Multiple ethnic groups, Other | **Categorical – white and Asian** | |
| What is your height? | (In Centimetres) | Continuous | |
| What is your weight? | (In Kilograms) | Continuous | |
| What sport are you currently playing? | Athletics, basketball, cycling, football, netball, lacrosse, rowing, hockey | Categorical  1 = contact sports (football, basketball, netball, lacrosse, hockey)  0 = non-contact sports (athletics, rowing, cycling) | |
| **Date of Testing** | **Game day -1, +1, +2, or +3** | **Categorical** | |
| How many minutes did you play in your match/competition? | (In minutes) | Continuous | |
| Have you eaten or drank today? | Yes/No | Categorical  No = 1  Yes = 0 | |
| Have you experienced any injury/illness in the past 7 days? | Yes/No | Categorical  Yes = 1  No = 0 | |
| Have you taken any NSAIDs in the past 24 hours? | Yes/No | Categorical | |
| Menstrual cycle symptoms: Lower back pain, Menstrual cramps, Bloating, nausea | Indicate severity: high, medium, low, not experiencing  (revisit depending on prevalence) | Categorical  Present = 1 (high or medium)^[[1]](#footnote-1)^  Absent = 0 (low or none)^4^ | |
| Fatigue symptoms: Fatigue/heavy legs/muscle ache/weak | Indicate severity: high, medium, low, not experiencing | Categorical  Present = 1 (high or medium)  Absent = 0 (low or none) | |

High or medium, and low or none were combined into present or absent, respectively due to a relatively low prevalence across all symptoms

| **Supplemental Table 2.** Baseline characteristics between the included and excluded sample | | | | |
| --- | --- | --- | --- | --- |
|  |  | **Included Sample (N=16)** | **Excluded Sample (N=4)** | **P-value** |
| **BASELINE QUESTIONNAIRE COVARIATES** |  |  |  |  |
| **Age** | Mean, SD | 21.30 ± 3.12 | 20.75 ± 0.96 | 0.50 |
| **BMI** | Mean, SD | 21.89 ± 1.54 | 21.94 ± 1.60 | 0.96 |
| **Ethnicity** |  |  |  | **0.03** |
| White | % (N) | 80.0% (14) | 25.0% (1) |  |
| Asian | % (N) | 10.0% (2) | 75.0% (3) |  |
| **Sport type**^1^ |  |  |  | 0.14 |
| Contact sports | % (N) | 87.5% (14) | 50.0% (2) |  |
| Non-contact sports | % (N) | 12.5% (2) | 50.0% (2) |  |
| **Injury within the last 3 months** |  |  |  |  |
| Yes^2^ | % (N) | 12.5% (2) | 25.0% (1) | 0.12 |
| No | % (N) | 87.5% (14) | 75.0% (3) |  |
| **GAME DAY COVARIATES** |  |  |  |  |
| **Fatigue^3^** |  |  |  | 0.26 |
| Yes | % (N) | 52.9% (63) | 38.5% (10) |  |
| No | % (N) | 47.1% (56) | 61.5% (16) |  |
| **Menstrual cycle symptoms^3^** |  |  |  | 0.26 |
| Yes | % (N) | 37.0% (44) | 23.1% (6) |  |
| No | % (N) | 63.0% (75) | 76.9% (20) |  |
| **Fasted^3^** |  |  |  | 0.71 |
| Yes | % (N) | 87.4% (104) | 92.3% (24) |  |
| No | % (N) | 12.6% (15) | 7.7% (2) |  |
| **Injury during the study^3^** |  |  |  | 0.15 |
| Yes | % (N) | 22.7% (27) | 38.5% (10) |  |
| No | % (N) | 87.3% (92) | 61.5% (16) |  |
| **Minutes played** | Mean, SD | 57.32 ± 16.72 | 52.86 ± 28.21 | 0.42 |

Contact sports – football, hockey, netball, lacrosse, basketball; non-contact sports – athletics, cycling, rowing.

^2^ The two injuries were a calf strain (unable to play for one week) and shoulder pain (able to play through injury).

3 Coded for as binary (1 = presence of symptoms/fasted state, 0 = no presence of symptoms/not fasted)

| **Supplemental Table 3.** Comparison of covariates during the study across each menstrual cycle phase (n=16 participants; n= 119 data collection days) | | | | | | |
| --- | --- | --- | --- | --- | --- | --- |
| **Covariate** |  | **Early Follicular (n=25)** | **Late Follicular (n=35)** | **Mid Luteal (n=39)** | **Late Luteal (n=20)** | **P-value** |
| **Fatigue^1^** |  |  |  |  |  | 0.67 |
| Yes | % (N) | 60.0% (15) | 57.1% (20) | 48.7% (19) | 45.0% (9) |  |
| No | % (N) | 40.0% (10) | 42.9% (15) | 51.3% (20) | 55.0% (11) |  |
| **Injured/illness^2^** |  |  |  |  |  | 0.42 |
| Yes | % (N) | 20.0% (5) | 28.6% (10) | 25.6% (10) | 10.0% (2) |  |
| No | % (N) | 80.0% (20) | 71.4% (25) | 74.4 (29) | 90.0% (18) |  |
| **Menstrual cycle symptoms^1^** |  |  |  |  |  | **<0.001** |
| Yes | % (N) | 68.0% (17) | 20.0% (7) | 20.5% (8) | 60.0% (12) |  |
| No | % (N) | 32.0% (8) | 80.0% (28) | 79.5% (31) | 40.0% (8) |  |
| **Fasted^1^** |  |  |  |  |  | 0.97 |
| Yes | % (N) | 88.0% (22) | 85.7% (30) | 87.2% (34) | 90.0% (18) |  |
| No | % (N) | 12.0% (3) | 14.3% (5) | 12.8% (5) | 10.0% (2) |  |
| **Minutes played** | Mean (SD) | 59.60 ± 20.50 | 53.60 ± 16.6 | 60.30 ± 15.30 | 62.20 ± 17.5 | 0.24 |

1 Coded for as binary (1 = presence of symptoms/fasted state, 0 = no presence of symptoms/not fasted)

^2^ The two injuries were a calf strain (unable to play for one week) and shoulder pain (able to play through injury).

| **Supplemental Table 4**. Interactions between menstrual cycle phases and game days (sample size = 16; 119 data collection days) | | | | | | | |
| --- | --- | --- | --- | --- | --- | --- | --- |
|  | **Unadjusted Model** | | |  | **Adjusted Model** | | |
| **Predictor** | **Estimate (B)** | **95% CI** | **P-value** |  | **Estimate (B)** | **95% CI** | **P-value** |
| GD-1 (reference) | - | - | - |  | - | - | - |
| GD+1 | 24.02 | 0.77 - 48.65 | 0.078 |  | 24.20 | -3.96 – 52.35 | 0.09 |
| GD+2 | 21.22 | -2.86 – 45.15 | 0.11 |  | 24.21 | -2.94 – 51.36 | 0.08 |
| GD+3 | -10.32 | -34.64 – 13.85 | 0.44 |  | -6.82 | -34.08 – 20.44 | 0.62 |
| Early Follicular (reference) | - | - | - |  | - | - | - |
| Late Follicular | -3.98 | -26.57 – 18.51 | 0.75 |  | -0.50 | -25.52 – 24.52 | 0.97 |
| Mid Luteal | 7.66 | -17.34 – 32.53 | 0.57 |  | 18.08 | -9.08 – 45.24 | 0.19 |
| Late Luteal | 0.96 | -23.91 – 25.65 | 0.94 |  | 4.09 | -23.20 – 31.39 | 0.77 |
| Interaction Terms |  |  |  |  |  |  |  |
| LF:GD+1 | -5.96 | -37.62 – 25.87 | 0.73 |  | -8.26 | -44.16 – 27.63 | 0.65 |
| ML:GD+1 | -23.36 | -54.99 – 8.45 | 0.18 |  | -25.47 | -61.41 – 10.48 | 0.16 |
| LL:GD+1 | 38.91 | 5.18 – 72.92 | **0.04** |  | 40.45 | 2.04 - 78.86 | **0.04** |
| LF:GD+2 | -5.39 | -36.19 – 25.59 | 0.75 |  | -11.17 | -45.75 – 23.41 | 0.52 |
| ML:GD+2 | -18.27 | -49.41 – 13.06 | 0.29 |  | -23.18 | -58.34 – 11.99 | 0.19 |
| LL:GD+2 | -22.50 | -58.02 – 13.32 | 0.25 |  | -25.3 | -65.18 – 14.58 | 0.21 |
| LF:GD+3 | 21.00 | -9.73 – 51.92 | 0.21 |  | 14.72 | -19.51 – 48.94 | 0.4 |
| ML:GD+3 | -1.68 | -33.42 – 30.25 | 0.92 |  | -8.53 | -44.07 – 27.02 | 0.64 |
| LL:GD+3 | 4.68 | -33.18 – 42.82 | 0.82 |  | 1.04 | -41.26 – 43.35 | 0.96 |

| **Supplemental Table 5.** High sensitivity C-reactive protein (hs-CRP) estimates across different menstrual cycle phases relative to game days, derived by re-pivoting the main model with each phase as the reference | | | | | | | | |
| --- | --- | --- | --- | --- | --- | --- | --- | --- |
|  |  | **Unadjusted model** | | |  | **Adjusted model** | | |
| **Predictor** |  | **Estimate (B)** | **95% CI** | **P-value** |  | **Estimate (B)** | **95% CI** | **P-value** |
| **Early follicular** |  |  |  |  |  |  |  |  |
| GD-1 (reference) |  | - | - | - |  | - | - | - |
| GD+1 |  | 24.02 | -0.78 – 48.65 | 0.08 |  | 24.20 | -3.96 – 52.35 | 0.09 |
| GD+2 |  | 21.22 | -2.86 – 45.15 | 0.11 |  | 24.21 | -2.94 – 51.36 | 0.08 |
| GD+3 |  | -10.32 | -34.64 – 13.85 | 0.44 |  | -6.82 | -34.08 – 20.44 | 0.62 |
| **Late follicular** |  |  |  |  |  |  |  |  |
| GD-1 (reference) |  | - | - | - |  | - | - | - |
| GD+1 |  | 18.06 | -1.84 – 37.96 | 0.10 |  | 15.93 | -6.45 – 38.32 | 0.16 |
| GD+2 |  | 15.83 | -3.39 – 35.07 | 0.14 |  | 13.04 | -8.47 – 34.55 | 0.23 |
| GD+3 |  | 10.68 | -7.89 – 29.28 | 0.30 |  | 7.90 | -12.89 – 28.69 | 0.45 |
| **Mid luteal** |  |  |  |  |  |  |  |  |
| GD-1 (reference) |  | - | - | - |  | - | - | - |
| GD+1 |  | 0.65 | -19.17 – 20.50 | 0.95 |  | -1.27 | -23.64 – 21.10 | 0.91 |
| GD+2 |  | 2.95 | -16.87 – 22.80 | 0.79 |  | -1.03 | -21.34 – 23.40 | 0.93 |
| GD+3 |  | -12.00 | -32.29 – 8.33 | 0.28 |  | -15.35 | -38.15 – 7.45 | 0.18 |
| **Late luteal** |  |  |  |  |  |  |  |  |
| GD-1 (reference) |  | - | - | - |  | - | - | - |
| GD+1 |  | 62.93 | 40.31 – 85.66 | **<0.001** |  | 64.65 | 38.80 – 90.49 | **<0.001** |
| GD+2 |  | -1.28 | -27.15 – 24.73 | 0.93 |  | -1.09 | -30.17 – 27.99 | 0.94 |
| GD+3 |  | -5.64 | -34.35 – 23.19 | 0.72 |  | -5.78 | -37.83 – 26.27 | 0.72 |


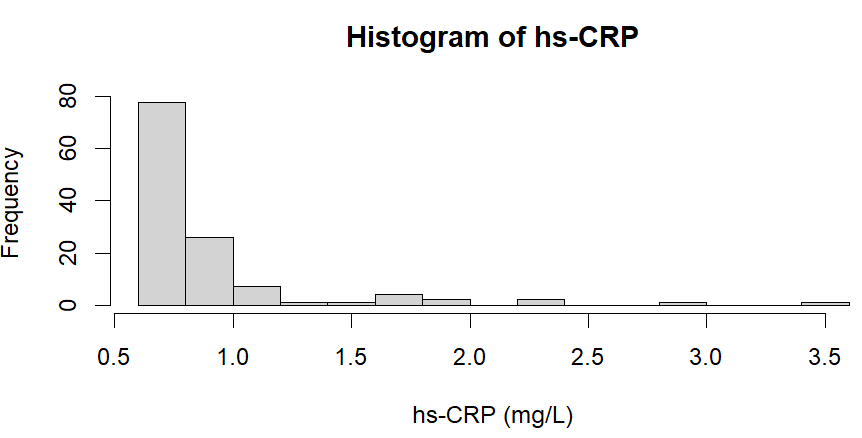
**Supplemental Figure 1.** Histogram showing the distribution of hs-CRP concentrations across the whole study period


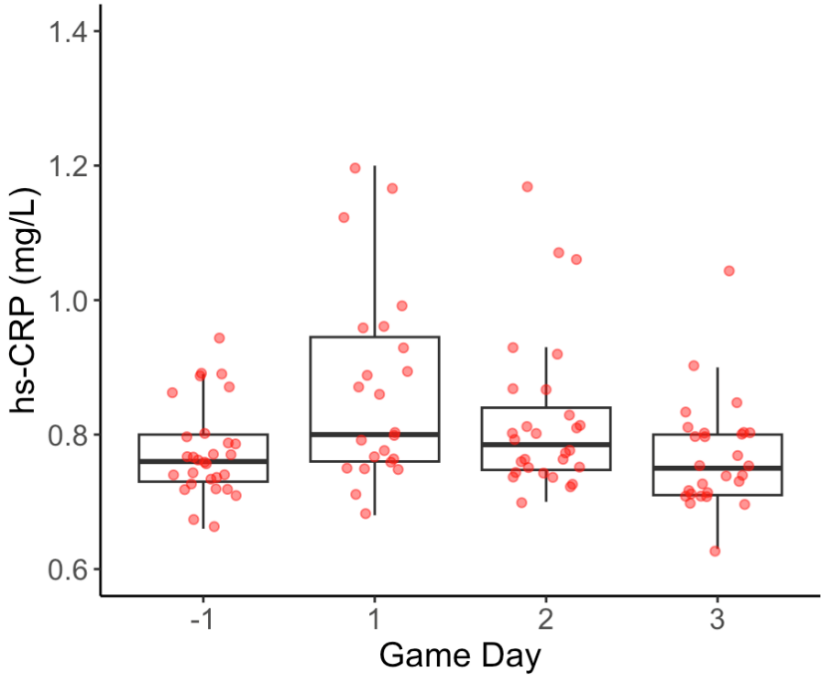


**Supplemental Figure 2 .** Boxplot showing the median hs-CRP (high-sensitivity C-reactive protein) concentrations seen across each game

1. [↑](#footnote-ref-1)
